# Greater Social Competence Is Associated With Higher Interpersonal Neural Synchrony in Adolescents With Autism

**DOI:** 10.3389/fnhum.2021.790085

**Published:** 2022-01-06

**Authors:** Alexandra P. Key, Yan Yan, Mary Metelko, Catie Chang, Hakmook Kang, Jennifer Pilkington, Blythe A. Corbett

**Affiliations:** ^1^Vanderbilt Kennedy Center, Vanderbilt University Medical Center, Nashville, TN, United States; ^2^Department of Hearing and Speech Sciences, Vanderbilt University Medical Center, Nashville, TN, United States; ^3^Vanderbilt University, Nashville, TN, United States; ^4^Institute for Software Integrated Systems, Vanderbilt University, Nashville, TN, United States; ^5^Department of Electrical and Computer Engineering, Vanderbilt University, Nashville, TN, United States; ^6^Department of Biostatistics, Vanderbilt University Medical Center, Nashville, TN, United States; ^7^Department of Psychiatry and Behavioral Sciences, Vanderbilt University Medical Center, Nashville, TN, United States

**Keywords:** autism, hyperscanning, EEG, social, synchrony, sex differences

## Abstract

Difficulty engaging in reciprocal social interactions is a core characteristic of autism spectrum disorder. The mechanisms supporting effective dynamic real-time social exchanges are not yet well understood. This proof-of-concept hyperscanning electroencephalography study examined neural synchrony as the mechanism supporting interpersonal social interaction in 34 adolescents with autism spectrum disorder (50% female), age 10–16 years, paired with neurotypical confederates of similar age. The degree of brain-to-brain neural synchrony was quantified at temporo-parietal scalp locations as the circular correlation of oscillatory amplitudes in theta, alpha, and beta frequency bands while the participants engaged in a friendly conversation. In line with the hypotheses, interpersonal neural synchrony was significantly greater during the social interaction compared to the baseline. Lower levels of synchrony were associated with increased behavioral symptoms of social difficulties. With regard to sex differences, we found evidence for stronger interpersonal neural synchrony during conversation than baseline in females with autism, but not in male participants, for whom such condition differences did not reach statistical significance. This study established the feasibility of hyperscanning during real-time social interactions as an informative approach to examine social competence in autism, demonstrated that neural coordination of activity between the interacting brains may contribute to social behavior, and offered new insights into sex-related variability in social functioning in individuals with autism spectrum disorders.

## Introduction

Coordination of behavior between individuals—social synchrony—is a fundamental aspect of social life (Koike et al., 2015) and a known area of weakness in autism spectrum disorder (ASD). ASD is characterized by impairments in social competence (American Psychiatric Association, 2013) and difficulties with reciprocal social interactions (Bauminger and Shulman, 2003). Previous work on social competence in ASD characterized discrete social information processing skills in individual participants (e.g., face perception, emotion recognition, etc.; Tsang et al., 2019; Griffin et al., 2020). However, due to the dynamic nature of real-life social interactions, the mechanisms supporting successful social engagement with others are not yet well understood.

The ability to objectively quantify the degree of connectedness among social partners during a naturalistic interaction offers a novel approach to study social functioning (Dikker et al., 2017). Studies in typical adults demonstrated that successful interactions involve alignment of behavior (Richardson et al., 2007; Konvalinka et al., 2010; Hale et al., 2019) and physiology (heart rate: Konvalinka et al., 2011; respiration: McFarland, 2001). Recent developments in simultaneous neuroimaging data acquisition—hyperscanning—make it possible to examine interbrain coordination while the subjects freely engage in a social task in natural settings (Kinreich et al., 2017; Pérez et al., 2017; Czeszumski et al., 2020). The emerging data (e.g., Dumas et al., 2011; Astolfi et al., 2012; Hasson et al., 2012) note that many forms of social behavior and cognition, such as the theory of mind, conversational turn-taking, and emotion regulation, involve interbrain synchrony across multiple frequency bands. The associated brain areas include posterior superior temporal sulcus (pSTS) and temporoparietal junction (TPJ; Dikker et al., 2014; Kinreich et al., 2017).

One of the most common forms of social interaction in daily life is a conversation with a partner (e.g., a family member, a friend, etc.). It involves processing both verbal and nonverbal cues in order to successfully maintain the overall flow and engagement. Difficulty engaging in reciprocal social interactions is a core characteristic of ASD (American Psychiatric Association, 2013). The utility of a conversation as a means to evaluate social skills in ASD is highlighted by its inclusion in formal assessment procedures, such as the Contextual Assessment of Social Skills (CASS; Ratto et al., 2011). Conversation skills are also a frequent treatment target in ASD (Nuernberger et al., 2013; Stewart Rosenfield et al., 2019). Thus, examining the coordination of neural activity in participants with ASD and their conversation partners may offer insights into the mechanisms supporting social engagement. Previously, in neurotypical adults, a hyperscanning study using electroencephalography during naturalistic conversations reported greater interbrain neural synchrony among socially connected (e.g., romantic partners) compared to stranger dyads (Kinreich et al., 2017). The specific content of the conversations did not affect the extent of synchrony. The observed interbrain associations also could not be attributed exclusively to physical properties of the speech signal or to motor artifacts from jaw movements: while lower EEG frequency bands (e.g., theta) did demonstrate entrainment to speech rhythm, increased synchrony in the higher frequencies (e.g., alpha, beta) was independent of specific speech characteristics (Pérez et al., 2017). Similarly, a hyperscanning study of typically developing high school students reported increased interpersonal neural synchrony in participants who were more focused on the shared activity, exhibited higher empathy skills, and reported greater social closeness with the partner, independent of the specific group activity (Dikker et al., 2017). The studies in neurotypical participants established hyperscanning as an effective means to quantify neural markers of interpersonal social engagement.

Data on interpersonal coordination during a real-time conversation in individuals with ASD are limited, but recent studies in adults suggest reduced motor synchrony (head and body movements) with dyadic partners (Georgescu et al., 2020) and reduced interpersonal neural synchrony in the TPJ compared to neurotypical dyads (Quiñones-Camacho et al., 2021), demonstrating the sensitivity of interpersonal synchrony measures to atypical social functioning.

Adolescence is the developmental period of increasing drive to engage in social interactions with peers and the corresponding maturational changes in the structure and function of the neural systems supporting social information processing, including the TPJ and pSTS (see Lamblin et al., 2017 for review). Therefore, adolescence could be a particularly informative time window for investigating social functioning in ASD. In typical development, sex differences in the goals for and approaches to social interaction also become apparent during adolescence, with females demonstrating more prosocial behaviors and greater concern for maintaining social connections than males (see Rose and Rudolph, 2006 for review). In ASD, the question of sex differences in social functioning is understudied, due in part to the diagnosis being more common in males, with a ratio of 4:1 (Maenner et al., 2020). However, the emerging findings suggest that ASD may be underdiagnosed in females (Kim et al., 2011; Loomes et al., 2017; Ratto et al., 2018) because their social difficulties are often less noticeable (Mandy et al., 2012; Dean et al., 2017) and could be effectively camouflaged (Dworzynski et al., 2012; Lai et al., 2015; Corbett et al., 2020).

The goal of this proof-of-concept study was to examine interpersonal synchrony as a neural correlate of the individual differences in social competence in adolescents with ASD. We hypothesized that the magnitude of neural synchronization with a social partner will: (1) increase during the active interaction compared to the baseline; and (2) correlate with autism symptom severity, caregiver reports of social functioning, and theory of mind skills. In addition, given recent evidence that females with ASD may be more successful than males at masking their social difficulties (Halladay et al., 2015; Corbett et al., 2020), we predicted greater interpersonal neural synchrony in females than males with ASD.

## Materials and Methods

### Participants

Thirty-four adolescents with ASD, age 10–16 years, participating in a randomized clinical trial of a social skills treatment (SENSE Theatre^®^; www.clinicaltrials.gov ID# NCT02276534) contributed EEG data for this study. The sample included all available females (*n* = 17) and 17 males matched on age and IQ (WASI; Wechsler, 1999). The diagnosis of ASD was made in accordance with the Diagnostic and Statistical Manual-5 (American Psychiatric Association, 2013) based on: a previous diagnosis by a professional with autism expertise; current clinical judgment (B.A.C.); and the Autism Diagnostic Observation Schedule (ADOS; Lord et al., 2000), administered to all participants by research-reliable personnel. Social Communication Questionnaire (SCQ; Rutter et al., 2003) further corroborated the diagnosis (scores ≥15). Males and females were not significantly different on standardized behavioral measures of IQ, social functioning, or theory of mind skills (NEPSY; Korkman et al., 2007). However, males with ASD had higher ADOS severity scores (Table 1). Parents/guardians of the participants provided written informed consent, and participants provided assent. The Institutional Review Board of Vanderbilt University Medical Center approved the study procedures.

**Table 1:** Demographic and clinical characteristics of adolescents with ASD included in the study.

	Females (*n* = 17)		Males (*n* = 17)		
	M	SD	M	SD	*p*-value
Age (years)	13.06	1.62	12.80	1.50	0.63
ADOS—Social Affect Total	8.18	3.40	10.06	3.98	0.15
ADOS—Restricted and Repetitive Behaviors Total	3.24	1.39	4.18	1.74	0.09
ADOS—Severity	6.76	1.56	8.00	1.50	0.03
WASI—Verbal	105.29	15.55	102.00	16.33	0.55
WASI—Performance	93.94	19.00	96.50	18.06	0.69
WASI—Full-scale IQ	99.29	17.16	99.94	18.23	0.92
SCQ (total)	17.18	8.99	17.12	7.11	0.98
NEPSY Theory of Mind—Verbal	18.24	3.60	17.18	5.26	0.50
NEPSY Theory of Mind—Contextual	4.71	1.05	4.35	1.32	0.39
NEPSY Theory of Mind—Total	22.94	4.26	21.53	5.80	0.43

### Procedure

Interpersonal neural synchrony was assessed using a hyperscanning protocol during which 128-channel EEG (Electrical Geodesics, Inc., Eugene, OR) was simultaneously and continuously recorded (sampling rate: 1,000 Hz) in each participant and an opposite-sex confederate of similar age during two blocks: a 3-min baseline (eyes-open resting EEG) with no overt verbal or nonverbal interaction, and a 5-min free-form conversation about a fun day in the past or the future (see Kinreich et al., 2017; Quiñones-Camacho et al., 2021 for similar naturalistic paradigms). The confederates were familiarized with the clinical presentation of ASD and were aware of the participants’ diagnostic status but blind to the hyperscanning study hypotheses regarding the interpersonal synchrony or sex differences. The confederates were trained to be friendly, demonstrate an interest in the conversation topic selected by the participant with ASD, and support the natural flow of the conversation while striving for an approximately equal time of speaking and listening. The inclusion of verbally proficient participants with ASD and the use of a specific topic for the conversation further reduced opportunities for misunderstanding or double-empathy problems that could arise when misperceptions by a neurotypical person create barriers for social participation by an autistic individual (e.g., Mitchell et al., 2021). The participants and confederates sat 1 m apart facing each other at a ~45° angle. The research staff provided instructions before each block and then left the room. The session was audio- and video-recorded for offline coding of social behaviors (Corbett et al., 2010; Kinreich et al., 2017).

In addition to the social interaction during the hyperscanning protocol, behavioral measures of social functioning also included caregiver reports on the Social Communication Questionnaire (SCQ; Rutter et al., 2003) and participants’ performance on NEPSY Theory of Mind (TOM) subtests (Korkman et al., 2007). The verbal portion (TOM-V) measured ability to understand false belief and figurative language, recognize mental states and imitation. The contextual portion (TOM-C) assessed the ability to relate emotions to social context. It required the participants to identify a picture that best represents the feelings of a character in six different scenarios. Both TOM-V and TOM-C subtest scores as well as the total score were used in the analyses.

### Data Analysis

#### Social Behavior

Social behavior of participants with ASD during the social interaction was scored using the criteria employed in a CASS assessment (Ratto et al., 2011), which is procedurally similar to the friendly conversation manipulation in the current study. The final data set included the number of questions asked or topic changes as well as 7-point Likert scale ratings of vocal expressiveness, frequency of gestures, positive affect, amount of physical movement, markers of social anxiety, overall interest in the conversation, and quality of the rapport. A trained researcher blind to the study hypotheses completed the ratings offline using video recordings of the conversations. Higher scores reflected more adaptive social behavior during the interaction.

#### EEG Data

Raw EEG recordings were divided into 3-min (baseline) and 5-min (interaction) segments, filtered at 0.1–30 Hz, referenced to the average reference, screened for motor artifacts using a custom automated Matlab pipeline adapted from Pérez et al. (2017) that removed segments contaminated by nonsystematic motor noise and used independent components analysis to correct systematic artifacts (e.g., eye blinks). The resulting artifact-free periods were time-matched within a dyad (baseline: *M* = 129.6 ± 23.64 s interaction: *M* = 43.25 ± 43.04 s) and submitted for statistical analyses. Following the approach of Kinreich et al. (2017) and Pérez et al. (2017), neural synchrony between the participants was quantified as the correlation of EEG oscillatory amplitudes in theta (4–8 Hz), alpha (8–12 Hz), and beta (12–30 Hz) bands. To reduce the likelihood of spurious hyperconnectivity results, the circular correlation (CCOR) metric was used (Burgess, 2013). Based on the recommended statistical approaches for hyperscanning data (Balconi and Vanutelli, 2017), the analysis focused on a subset of 12 temporoparietal electrodes in each hemisphere (left: 51, 52, 53, 54, 58, 59, 60, 61, 64, 65, 66, 67; right: 78, 79, 80, 85, 86, 87, 91, 92, 93, 96, 97, 98) rather than on all possible electrode pairs. The CCOR values within a dyad were calculated for each electrode pair in each frequency band, then averaged across the locations within each cluster. Preliminary analyses identified no hemisphere differences; therefore, CCOR values were averaged across left and right temporoparietal locations, further reducing the number of experimental factors.

For each frequency band, paired t-tests compared CCOR values to evaluate differences in interpersonal synchronization between the baseline and social interaction periods in the combined sample and within females and males with ASD separately. Group differences in interpersonal neural synchrony between female and male participants with ASD were examined using independent group t-tests. Brain-behavior associations were explored between changes in neural synchrony (differences in CCOR values between interaction vs. baseline periods) and standardized measures of autism, IQ, social functioning, and theory of mind. Given the directional predictions that greater interpersonal neural synchrony would be associated with fewer autism symptoms (lower ADOS Social Affect and severity scores), better social functioning (lower SCQ scores), and greater theory of mind skills (higher TOM scores), the significance of observed correlations in this exploratory analysis was evaluated using a 1-tail test.

According to the estimates from G*Power (v. 3.1.9.2), the study sample of 34 provided 80% statistical power to detect medium effect sizes (*d* = 0.5 or greater) for changes from baseline to active conversations, while each sex group (*n* = 17) was powered to detect effects of *d* = 0.65. For between-group differences, large effect sizes (*d* = 0.8) could be detected.

## Results

**Figure 1:**
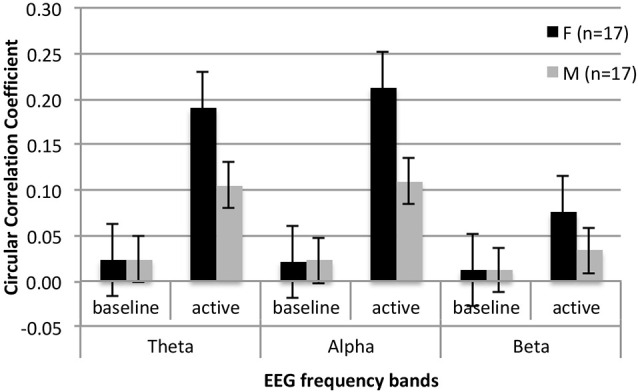
Mean circular correlation values for the temporoparietal clusters in females and males with ASD during baseline and active interaction conditions in three analyzed frequency bands.

Summary of the behavioral performance during the social interaction is presented in Table 2. No sex differences reached significance for any of the measures. Average CCOR values for baseline and social interaction conditions in each frequency band are summarized in Table 3. In the combined sample, there was a significant increase in neural synchrony (larger CCOR values) during the interaction compared to the baseline in each frequency band: theta *t*_(33)_ = 2.95, *p* = 0.006, *d* = 0.51; alpha *t*_(33)_ = 2.77, *p* = 0.009, *d* = 0.48; beta *t*_(33)_ = 3.13, *p* = 0.004, *d* = 0.54. Within-group follow-up analyses noted that the observed results were primarily driven by the female participants (Figure 1): theta *t*_(16)_ = 2.630, *p* = 0.018, *d* = 0.64, alpha *t*_(16)_ = 2.336, *p* = 0.033, *d* = 0.57, beta *t*_(16)_ = 2.599, *p* = 0.019, *d* = 0.63. In males, increased interpersonal synchrony in the beta band approached significance, *t*_(16)_ = 2.068, *p* = 0.055, *d* = 0.50, while no differences were significant for the other frequency bands. However, between-group sex-related differences did not reach significance for any of the frequency bands.

**Table 2:** Summary scores for behavioral performance during the social interaction for female and male participants with ASD.

	**Females**		**Males**		
	M	SD	M	SD	*p*-value
Questions (number)	3.29	3.41	3.06	3.34	0.84
Topic changes (number)	2.24	1.75	2.88	3.62	0.51
Vocal expressiveness	3.71	1.96	3.65	1.58	0.92
Gestures	2.88	1.41	2.71	1.45	0.72
Positive affect	3.82	2.13	2.71	1.49	0.09
Motor arousal	2.76	1.25	2.71	0.99	0.88
Social anxiety	2.82	0.95	2.47	1.23	0.36
Overall interest	3.88	1.36	3.47	1.33	0.38
Overall rapport	4.06	1.34	3.82	1.29	0.61

**Table 3:** Circular correlation (CCOR) values for theta, alpha, and beta bands in the left and right temporoparietal scalp clusters for female and male participants with ASD during the baseline and social interaction conditions.

	Female (*n* = 17)				Male (*n* = 17)				Total			
	Baseline		Social interaction		Baseline		Social interaction		Baseline		Social interaction	
	M	SD	M	SD	M	SD	M	SD	M	SD	M	SD
theta_left	0.024	0.009	0.184	0.269	0.023	0.008	0.097	0.199	0.023	0.008	0.140	0.237
theta_right	0.022	0.008	0.196	0.302	0.025	0.010	0.113	0.257	0.024	0.009	0.154	0.279
alpha_left	0.021	0.007	0.188	0.311	0.024	0.009	0.110	0.233	0.022	0.009	0.149	0.274
alpha_right	0.023	0.007	0.237	0.374	0.021	0.009	0.108	0.236	0.022	0.008	0.173	0.315
beta_left	0.013	0.005	0.066	0.076	0.012	0.005	0.035	0.049	0.012	0.005	0.051	0.065
beta_right	0.011	0.004	0.084	0.125	0.012	0.005	0.032	0.038	0.012	0.004	0.058	0.095

**Figure 2:**
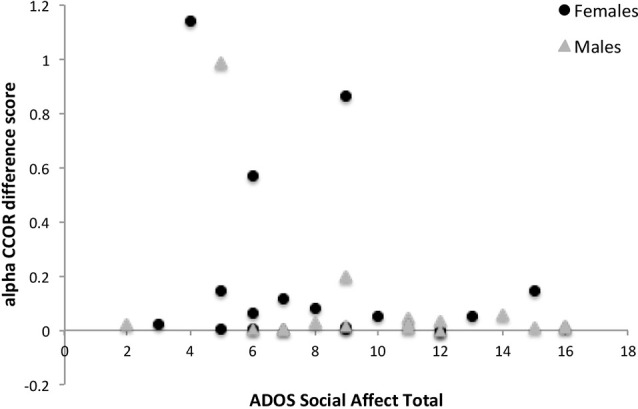
The relationship between ADOS Social Affect total scores and the difference in circular correlation values across baseline and active interaction conditions in the alpha band for females and males with ASD. ADOS, Autism Diagnostic Observation Schedule.

Results of the exploratory correlations between the measures of neural synchrony and individual differences in the clinical characteristics for the combined sample are presented in Table 4. The increase in interpersonal synchrony did not correlate with age or IQ. Conversely, the predicted association between greater interpersonal synchrony and lower ADOS Social Affect (Figure 2) and severity scores were observed. There was also a modest (*p* < 0.10) association between greater synchrony during the social interaction and higher TOM scores. Furthermore, correlations between neural measures and behavior during the social interaction revealed that a greater increase in interpersonal neural synchrony in the theta band correlated with greater vocal expressiveness (*r* = 0.34, *p* = 0.023) and more adaptive gesture use (*r* = 0.31, *p* = 0.037), while higher values in the beta band were associated with increased nonverbal displays of positive affect (*r* = 0.31, *p* = 0.036).

**Table 4:** Brain-behavior correlations between demographic characteristics, standardized behavioral assessments, and increase in CCOR values for active social interaction relative to baseline conditions.

	Combined sample (*n* = 34)		
	theta_diff	alpha_diff	beta_diff
Age	0.06	0.04	0.01
WASI—Verbal	0.12	0.17	0.21
WASI—Performance	−0.04	0.05	0.16
WASI—Full-scale IQ	0.03	0.11	0.19
ADOS—Social Affect Total	−0.33*	−0.32*	−0.30*
ADOS—Restricted Repetitive Behaviors Total	−0.08	−0.03	−0.09
ADOS—Severity	−0.40**	−0.37*	−0.36*
SCQ (total)	−0.27^#^	−0.30*	−0.24^#^
NEPSY TOM—Verbal	0.22^#^	0.22	0.25^#^
NEPSY TOM—Contextual	0.25^#^	0.26^#^	0.27^#^
NEPSY TOM—Total	0.26^#^	0.25^#^	0.28^#^

**p < 0.01, *p < 0.05, ^#^*p* < 0.1.

## Discussion

The study examined individual differences in interpersonal neural synchrony during a naturalistic social interaction as a possible correlate of social competence in adolescents with ASD. In line with the hypotheses, brain-to-brain synchrony in theta, alpha, and beta frequency bands increased between the baseline and active social engagement periods. The observed changes cannot be attributed purely to the shift from sitting quietly next to another person to participating in a conversation. The latter creates a shared activity that provides opportunities for common affect, anticipation of specific content, and prediction of changes (e.g., pauses in speech) that could drive brain-to-brain synchrony. Prior hyperscanning studies during a conversation in neurotypical participants related synchronization in the lower frequencies to entrainment to physical features of the speech signal (e.g., important for effective turn-taking) while coordinated activity in the higher frequencies was associated with general attention and mutual interaction independent of speech characteristics (Pérez et al., 2017, 2019; Kawasaki et al., 2013) and varied based on the degree of social connectedness within a dyad (stranger vs. partner; Kinreich et al., 2017). As predicted, in our sample of participants with ASD, a greater increase in interpersonal synchrony during the conversation period was associated with fewer clinical symptoms in the social domain and lower severity of autism. Conversely, no correlations were observed between the extent of neural synchrony and age or IQ.

The exploratory question addressed whether interpersonal neural synchrony in ASD may manifest differently in males and females. The combined sample demonstrated increased neural synchrony when interacting with a peer compared to the baseline. Within-group analyses revealed evidence for stronger interpersonal neural synchrony during conversation than baseline in females with autism, but not in male participants, for whom such condition differences did not reach statistical significance. These results provide preliminary insights into the possible mechanisms by which females with ASD succeed in camouflaging their social difficulties (Dean et al., 2017). For example, compared to autistic males, they may be more attuned to verbal and nonverbal signs provided by their interaction partners, and the associated increase in brain-to-brain synchrony supports the development and implementation of compensatory strategies, such as imitation (Gould and Ashton-Smith, 2011; Tierney et al., 2016).

The observed sex-related variability in neural synchrony could not be attributed to differences in physical behavior during the study. There were no group differences in the number or quality of motor events (gestures, overall movement levels) or in the number of questions asked and topic changes. In addition, males and females with ASD demonstrated similar behavioral levels of interest and rapport. Furthermore, although females had slightly lower ADOS severity scores, the two groups were not significantly different on the metrics of social functioning (ADOS Social Affect, SCQ) that would be relevant for interpreting the result of a social interaction paradigm. Finally, while we did not collect eye tracking data in the current study, possible differences in gaze distribution on facial features between males and females with ASD (Harrop et al., 2019) were ruled out as the reason for the observed sex differences because prior studies in typical populations reported increased interpersonal neural synchrony during a shared verbal exchange even when participant faces were not visible (Pérez et al., 2017, 2019).

Overall, our findings suggest that EEG hyperscanning during a naturalistic social interaction in adolescents with ASD is a feasible and informative approach to investigate individual differences in social competence. Neural measures of interpersonal synchrony will complement and expand the findings from the studies examining behavioral/motor synchrony (e.g., Georgescu et al., 2020), especially in participants with more limited motor repertoire (e.g., due to sensorimotor difficulties or immaturity). The direction of causal associations between motor and neural synchrony has not yet been clearly established. Increased motor synchrony (e.g., coordination of posture, gaze, gestures) is frequently observed among interacting neurotypical individuals and can be predictive of better social outcomes (Shockley et al., 2009; Zampella et al., 2020). Conversely, participants with ASD demonstrate reduced spontaneous motor synchronization with a social partner (Fitzpatrick et al., 2016; Zampella et al., 2020; Kruppa et al., 2021), suggesting that individual differences in motor control may interfere with interpersonal synchrony (McNaughton and Redcay, 2020). Although neural differences often precede behavioral symptoms (e.g., Dickinson et al., 2021), the neural mechanisms underlying atypical motor synchrony in individuals with ASD are poorly understood (Kruppa et al., 2021). Deficits in fine and gross motor skills and postural control (Fournier et al., 2010; Bhat et al., 2011; Kaur et al., 2018), as well as lower sensitivity and decreased attention to the movements of others (Fitzpatrick et al., 2016) have been reported in ASD. At the same time, Kruppa et al. (2021) observed no significant differences on the neural level between participants with ASD and neurotypical peers despite a reduction in their behavioral synchrony, and controlling for motor skills did not eliminate differences in interpersonal synchrony (Brezis et al., 2017). Conversely, Novembre et al. (2017) demonstrated that deliberately increasing neural synchrony (*via* transcranial stimulation) resulted in greater motor synchrony on a finger tapping task in neurotypical participants. Thus, future research will need to characterize the discrete contributions of motor coordination and interpersonal neural synchrony to social functioning.

Nevertheless, this proof-of-concept study has several limitations. The largely unstructured conversation could be considered a limitation due to the lack of control about the content or the time spent speaking vs. listening. However, previous studies have already established that interbrain synchrony reflects social connectedness independent of the conversation topic, specific spoken content, or the combination of periods of speaking and listening (Kinreich et al., 2017; Quiñones-Camacho et al., 2021). In real life, conversations with peers happen multiple times a day, making it a highly familiar experience that could be recreated in research settings with minimal disruptions to its natural form. The presence of trained confederates instructed to support the social interaction could have potentially biased the results toward increased interpersonal neural synchrony. However, this design choice was motivated by the desire to ensure consistent implementation of the experimental manipulation (a friendly conversation; see Quiñones-Camacho et al., 2021 for similar reasoning) as well as to facilitate a positive experience for the participants with ASD. Including naïve neurotypical individuals could have a similar confounding effect but in the opposite direction (i.e., reduced synchrony), as prior studies raised concerns about double empathy problems where neurotypical individuals inadvertently misperceive behaviors and/or verbal statements of a person with ASD leading to exaggerated social difficulties (e.g., Mitchell et al., 2021). Another limitation is the relatively small sample sizes for males and females with ASD that provided sufficient statistical power to detect only large effect sizes for between-group comparisons. Follow-up studies with larger samples will need to replicate the current findings. A related issue is the amount of data loss due to artifacts in the active condition. Motor noise due to speaking presents a challenge for EEG processing, resulting in reduced amounts of artifact-free signal during the conversation compared to the baseline periods, especially after matching temporal intervals with usable data between the participants within each dyad. Basing the analysis on the mean rather than peak CCOR values within the baseline and interaction periods and averaging data across electrode clusters rather than examining synchrony for the single electrodes was expected to minimize the effect of variable data loss on the results (Luck, 2005). Also, while the participants had full control of their conversation topic and flow, we did instruct them to remain seated and minimize gross body movements with the goal of maximizing data quality and reducing random effects on electrophysiological recordings. This limited our ability to analyze behavioral evidence of interpersonal coordination (e.g., head nods or facial and body movements—see Hale et al., 2019; Georgescu et al., 2020 for examples). Previously, motor synchrony has been identified as a possible contributor to social functioning (Shockley et al., 2009; Zampella et al., 2020). However, increased neural synchrony during a social interaction compared to the baseline has been previously observed even when the members of the dyad could not see each other (Pérez et al., 2017; Ahn et al., 2018). These results suggest that in addition to motor coordination, higher order cognitive processes (e.g., the ability to predict the other’s behavior) may contribute to differences in interpersonal neural synchrony (Kruppa et al., 2021). Furthermore, the findings from prior studies suggest that interpersonal synchrony measures may be relatively robust to momentary effects such as the content of the conversation, or amount of time speaking and listening (Kinreich et al., 2017; Pérez et al., 2017). While more data is always desirable, published network-level EEG analyses (e.g., microstates) report that reliable findings can be obtained with as few as 20 s of data (e.g., Koenig et al., 2002). The minimum sufficient data quantity for hyperscanning studies is yet to be established. In the meantime, novel EEG signal cleaning procedures for addressing speech-related motor artifacts are starting to emerge (e.g., Riès et al., 2021) and may support higher data retention rates in social interaction paradigms. Future work capitalizing on these developments as well as on ecological validity of real-time, face-to-face reciprocal social interactions and high temporal resolution of EEG will also need to investigate more fine-grained moment-to-moment associations between various social actions (e.g., eye contact, positive affect, head nods) and the neural coordination of activity between the interacting brains to more precisely characterize the mechanisms underlying social behaviors, the contributions of sex differences, and identify specific factors that facilitate or impede social synchrony. The resulting data will transform the way in which we think of and study typical and atypical social functioning.

## Data Availability Statement

The raw data supporting the conclusions of this article will be made available by the authors, without undue reservation.

## Ethics Statement

The studies involving human participants were reviewed and approved by Vanderbilt University Institutional Review Board. Written informed consent to participate in this study was provided by the participants’ legal guardian/next of kin.

## Author Contributions

AK and BC contributed to the conception of the study and critically revised the manuscript. AK collected data, performed statistical tests and functional interpretation of the results, wrote the draft of the manuscript. MM, CC, and YY provided expertise on designing and implementing the EEG processing and analysis pipeline. YY performed pre-processing of the EEG data, derived interpersonal neural synchrony metrics, and performed statistical tests. HK provided expertise in biostatistics methods. JP performed scoring of behavioral data. BC provided expertise on autism spectrum disorders and the interpretation of results. All authors contributed to the article and approved the submitted version.

## Conflict of Interest

The authors declare that the research was conducted in the absence of any commercial or financial relationships that could be construed as a potential conflict of interest.

## Publisher’s Note

All claims expressed in this article are solely those of the authors and do not necessarily represent those of their affiliated organizations, or those of the publisher, the editors and the reviewers. Any product that may be evaluated in this article, or claim that may be made by its manufacturer, is not guaranteed or endorsed by the publisher.
